# Supplementary material for: Complexes Between Adamantane Analogues B4X6 -X = {CH2, NH, O ; SiH2, PH, S} - and Dihydrogen, B4X6:nH2 (n = 1–4)
Source: Molecules. 2020 Feb 26;25(5):1042. doi: 10.3390/molecules25051042 (PMC7179137; doi:10.3390/molecules25051042)
Supplement: Supplementary file 1 [file molecules-25-01042-s001.pdf]

SUPPLEMENTARY INFORMATION

Figure 1. Plot of ΔE (kJ/mol) versus *d* (Å) with for the complex $[B_4(CH_2)_6]\cdots H_2$ for a range of distances 1.25 Å ≤ *d* ≤ 4.0 Å and a step Δ*d* = 0.25 Å. MP2/aug-cc-pVDZ (red solid line) and MP2/aug-cc-pVTZ (green dashed line) computations.

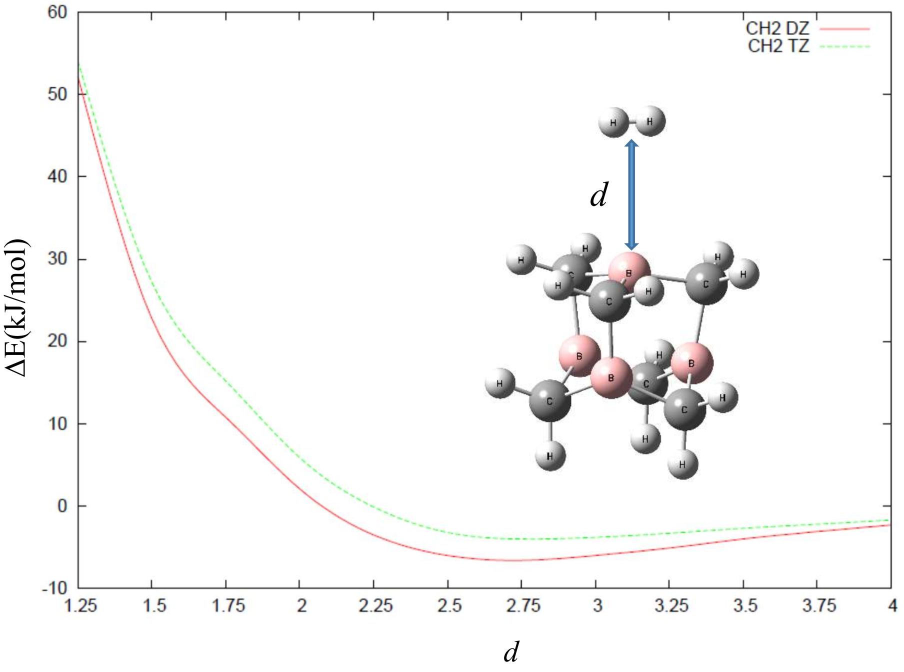

Figure 2. Optimized structures of the B_4X_6:$n$$H_2$ complexes (n = 1-4), X = {CH_2 ; NH, PH; S} with MP2/aug-cc-pVDZ computations. All geometries corrrespond to energy minima.

CARBON

(1:1) (1:2) (1:3) (1:4)

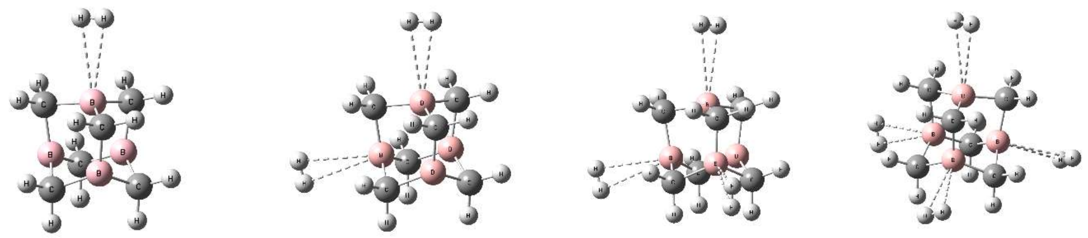

NITROGEN

(1:1) (1:2) (1:3) (1:4)

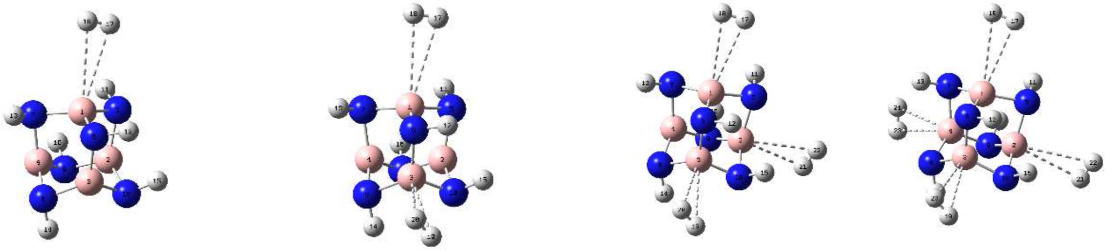

PHOSPHORUS

(1:1) (1:2) (1:3) (1:4)

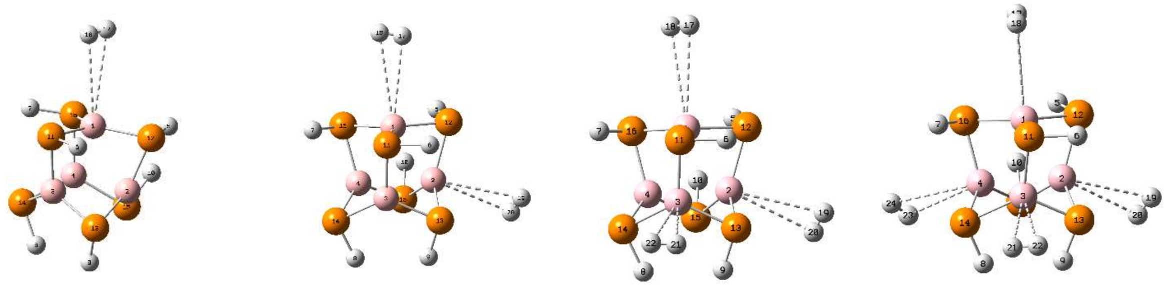

SULPHUR

(1:1) (1:2) (1:3) (1:4)

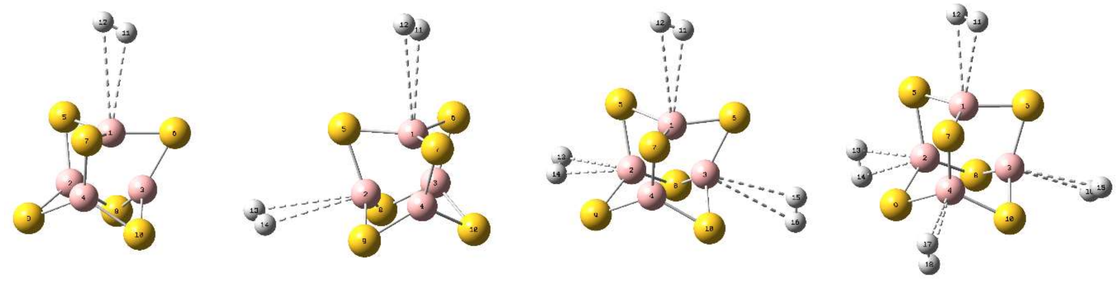

Table 1. Cartesian coordinates (Å) of MP2/aug-cc-pVDZ (left column) and MP2/aug-cc-pVTZ (right column) optimized geometries corresponding to energy minima of the adamantane analogue systems B_4X_6 with X = {CH_2, SiH_2; NH, PH; O, S}. All molecules have T_d symmetry except B_4X_6 with X = {NH, PH} which have C_1 symmetry.

$B_4(CH_2)_6$ (MP2/aug-cc-pVDZ) E = -334.198263 au				$B_4(CH_2)_6$ (MP2/aug-cc-pVTZ) E = -334.498131 au			
Atom	X	Y	Z	Atom	X	Y	Z
B	-1.136610	0.656222	-0.464019	B	-1.126811	0.650564	-0.460018
B	0.000000	0.000000	1.392057	B	0.000000	0.000000	1.380056
B	0.000000	-1.312444	-0.464019	B	0.000000	-1.301129	-0.460018
B	1.136610	0.656222	-0.464019	B	1.126811	0.650564	-0.460018
C	0.000000	-1.573495	1.112629	C	0.000000	-1.562461	1.104827
C	-1.362686	0.786747	1.112629	C	-1.353131	0.781230	1.104827
C	-1.362686	-0.786747	-1.112629	C	-1.353131	-0.781230	-1.104827
C	1.362686	0.786747	1.112629	C	1.353131	0.781230	1.104827
C	0.000000	1.573495	-1.112629	C	0.000000	1.562461	-1.104827
C	1.362686	-0.786747	-1.112629	C	1.353131	-0.781230	-1.104827
H	0.913880	-2.074432	1.466845	H	0.904696	-2.057529	1.454893
H	-0.913880	-2.074432	1.466845	H	-0.904696	-2.057529	1.454893
H	-2.253451	0.245772	1.466845	H	-2.234221	0.245274	1.454893
H	-1.339571	1.828660	1.466845	H	-1.329524	1.812254	1.454893
H	-1.339571	-0.773401	-2.213025	H	-1.329524	-0.767601	-2.193574
H	-2.253451	-1.301030	-0.720665	H	-2.234221	-1.289928	-0.716211
H	1.339571	-0.773401	-2.213025	H	1.329524	-0.767601	-2.193574
H	2.253451	-1.301030	-0.720665	H	2.234221	-1.289928	-0.716211
H	2.253451	0.245772	1.466845	H	2.234221	0.245274	1.454893
H	1.339571	1.828660	1.466845	H	1.329524	1.812254	1.454893
H	0.000000	1.546803	-2.213025	H	0.000000	1.535203	-2.193574
H	0.000000	2.602061	-0.720665	H	0.000000	2.579856	-0.716211
$B_4(SiH_2)_6$ (MP2/aug-cc-pVDZ) E = -1840.120753 au				$B_4(SiH_2)_6$ (MP2/aug-cc-pVTZ) E = -1840.397176 au			
Atom	X	Y	Z	Atom	X	Y	Z
B	-1.426555	0.823622	-0.582388	B	-1.413585	0.816133	-0.577093
B	0.000000	0.000000	1.747166	B	0.000000	0.000000	1.731281
B	0.000000	-1.647244	-0.582388	B	0.000000	-1.632267	-0.577093
B	1.426555	0.823622	-0.582388	B	1.413585	0.816133	-0.577093
H	1.224976	-2.707777	1.914687	H	1.217149	-2.685999	1.899288
H	-1.224976	-2.707777	1.914687	H	-1.217149	-2.685999	1.899288
H	-2.957491	0.293027	1.914687	H	-2.934718	0.288916	1.899288
H	-1.732515	2.414749	1.914687	H	-1.717568	2.397082	1.899288
H	-1.732515	-1.000268	-2.914876	H	-1.717568	-0.991638	-2.893087
H	-2.957491	-1.707508	-0.914498	H	-2.934718	-1.694360	-0.905489
H	1.732515	-1.000268	-2.914876	H	1.717568	-0.991638	-2.893087
H	2.957491	-1.707508	-0.914498	H	2.934718	-1.694360	-0.905489
H	2.957491	0.293027	1.914687	H	2.934718	0.288916	1.899288
H	1.732515	2.414749	1.914687	H	1.717568	2.397082	1.899288
H	0.000000	2.000536	-2.914876	H	0.000000	1.983277	-2.893087
H	0.000000	3.415017	-0.914498	H	0.000000	3.388721	-0.905489
Si	0.000000	-2.010143	1.421386	Si	0.000000	-1.993206	1.409410
Si	-1.740835	1.005071	1.421386	Si	-1.726167	0.996603	1.409410
Si	1.740835	1.005071	1.421386	Si	1.726167	0.996603	1.409410
Si	0.000000	2.010143	-1.421386	Si	0.000000	1.993206	-1.409410
Si	1.740835	-1.005071	-1.421386	Si	1.726167	-0.996603	-1.409410
Si	-1.740835	-1.005071	-1.421386	Si	-1.726167	-0.996603	-1.409410
$B_4(NH)_6$ (MP2/aug-cc-pVDZ) E = -430.581755 au				$B_4(NH)_6$ (MP2/aug-cc-pVTZ) E = -430.950451 au			
Atom	X	Y	Z	Atom	X	Y	Z

B	0.428828	0.868120	0.867229
B	-0.255164	0.543985	-1.139263
B	0.914321	-0.931094	-0.127376
B	-1.113359	-0.567493	0.437212
N	0.196881	1.739837	-0.349048
N	1.610113	-0.038300	0.871548
N	-0.759737	0.331365	1.577572
N	-0.259514	-1.759171	0.271759
N	-1.632776	0.034563	-0.828728
N	0.833906	-0.426760	-1.529083
H	-0.592499	2.363101	-0.154899
H	2.384061	0.434506	0.394590
H	-0.443606	-0.235475	2.369867
H	-0.569568	-2.271185	-0.559643
H	1.656990	0.141025	-1.752872
H	-2.230615	0.829700	-0.584192

B	-0.421400	-0.865243	0.864084
B	0.251968	-0.539565	-1.133825
B	-0.913615	0.923466	-0.123734
B	1.108205	0.568894	0.431742
N	-0.187641	-1.728615	-0.345029
N	-1.598188	0.031577	0.868567
N	0.759607	-0.324822	1.565457
N	0.253250	1.748699	0.267159
N	1.620004	-0.030222	-0.827448
N	-0.836653	0.419261	-1.515463
H	0.596001	-2.348049	-0.156128
H	-2.370516	-0.437844	0.402357
H	0.450806	0.233107	2.357143
H	0.556154	2.264014	-0.555404
H	-1.652534	-0.144890	-1.739629
H	2.221658	-0.815238	-0.592380

$B_4(PH)_6$ (MP2/aug-cc-pVDZ) E =-2147.787104 au

Atom	X	Y	Z
B	1.099006	-0.667265	0.795708
B	0.693220	0.833045	-0.970552
B	-0.885016	-1.006592	-0.629905
B	-0.890524	0.813190	0.868851
H	2.610400	1.385865	0.587744
H	1.216579	-2.485568	-0.999629
H	-0.631917	-0.949429	2.714271
H	-2.883374	0.555435	-0.704491
H	-1.367731	0.630138	-2.532482
H	0.753595	2.806646	0.646892
P	0.373915	-2.302960	0.143231
P	2.421837	0.267180	-0.281046
P	-0.299802	-0.290715	-2.297989
P	-2.408852	-0.271275	0.359987
P	-0.264175	2.328422	-0.234719
P	0.191678	0.149015	2.308349

$B_4(PH)_6$ (MP2/aug-cc-pVTZ) E = -2148.103969 au

Atom	X	Y	Z
B	1.068570	-0.703575	0.768142
B	0.717337	0.852451	-0.891808
B	-0.875588	-0.946149	-0.675723
B	-0.892725	0.766889	0.870200
H	2.594540	1.320397	0.702885
H	1.199837	-2.420571	-1.097570
H	-0.697645	-1.074385	2.616559
H	-2.849685	0.609581	-0.727802
H	-1.294373	0.762973	-2.501773
H	0.759509	2.740759	0.791852
P	0.351986	-2.281298	0.031728
P	2.418531	0.255563	-0.213605
P	-0.256854	-0.176039	-2.281994
P	-2.406438	-0.257961	0.299716
P	-0.227963	2.314500	-0.128975
P	0.134063	0.026113	2.283916

B_4O_6 (MP2/aug-cc-pVDZ) E = -549.777243 au

Atom	X	Y	Z
B	0.000000	0.000000	1.262242
B	-1.030616	0.595026	-0.420747
B	1.030616	0.595026	-0.420747
B	0.000000	-1.190053	-0.420747
O	-1.212041	0.699772	0.989627
O	1.212041	0.699772	0.989627
O	0.000000	-1.399544	0.989627
O	0.000000	1.399544	-0.989627
O	-1.212041	-0.699772	-0.989627
O	1.212041	-0.699772	-0.989627

B_4O_6 (MP2/aug-cc-pVTZ) E = -550.250527 au

Atom	X	Y	Z
B	0.000000	0.000000	1.257649
B	-1.026866	0.592861	-0.419216
B	1.026866	0.592861	-0.419216
B	-0.000000	-1.185722	-0.419216
O	-1.199450	0.692503	0.979347
O	1.199450	0.692503	0.979347
O	0.000000	-1.385006	0.979347
O	0.000000	1.385006	-0.979347
O	-1.199450	-0.692503	-0.979347
O	1.199450	-0.692503	-0.979347

B_4S_6 (MP2/aug-cc-pVDZ) E = -2485.309501 au

Atom	X	Y	Z
B	0.000000	0.000000	1.255644
B	-1.025229	0.591916	-0.418548
B	1.025229	0.591916	-0.418548
B	0.000000	-1.183832	-0.418548

B_4S_6 (MP2/aug-cc-pVTZ) E = -2485.678123 au

Atom	X	Y	Z
B	0.000000	0.000000	1.252465
B	-1.022633	0.590417	-0.417488
B	1.022633	0.590417	-0.417488
B	0.000000	-1.180835	-0.417488

S	-1.583624	0.914305	1.293023	S	-1.564782	0.903427	1.277639
S	1.583624	0.914305	1.293023	S	1.564782	0.903427	1.277639
S	0.000000	-1.828611	1.293023	S	0.000000	-1.806855	1.277639
S	0.000000	1.828611	-1.293023	S	0.000000	1.806855	-1.277639
S	-1.583624	-0.914305	-1.293023	S	-1.564782	-0.903427	-1.277639
S	1.583624	-0.914305	-1.293023	S	1.564782	-0.903427	-1.277639
